# Diversity and Drug Resistance of Filamentous Fungi Isolated from the Fresh Raspberries

**DOI:** 10.1007/s12088-021-00966-y

**Published:** 2021-08-15

**Authors:** Ewelina Farian, Angelina Wójcik-Fatla

**Affiliations:** Department of Health Biohazards and Parasitology, Institute of Rural Health, Jaczewskiego 2, 20-090 Lublin, Poland

**Keywords:** Filamentous fungi, Drug resistance, Food safety, Raspberries

## Abstract

Fungi are one of the most widely distributed microorganisms in the environment, including food such as fruits, vegetables and other crops, posing a potential threat to food safety and human health. The aim of this study was to determine the diversity, intensity and drug resistance of potentially pathogenic filamentous fungi isolated from the fresh raspberries (*Rubus idaeus* L.). A total of 50 strains belonging to genera *Fusarium*, *Cladosporium*, *Alternaria*, *Penicillium*, *Mucor*, *Rhizopus*, *Aspergillus* and *Acremonium* were tested for drug resistance against 11 antifungals by disc diffusion and gradient strips methods. The average mycological contamination in the examined samples of raspberries amounted to 4.34 log CFU/g. The *Cladosporium* was isolated from all tested samples, followed by *Alternaria* and *Fusarium* with a frequency of 61% and 34%, respectively. The highest level of drug resistance was observed for *Acremonium* genera and *Fusarium* strains recorded a wide variation in drug resistance as revealed by susceptibility with amphotericin B and voriconzole with MICs ranged from 0.5–4 µg/ml and posaconazole with MICs ranging from 3–8 µg/ml. All fungal strains showed 100% resistance to caspofungin, fluconazole and flucytosine with both the methods, and 100% resistance to micafungin and anidulafungin in the gradient strip method.

Fresh fruit play a pivotal role in human nutrition as an essential component of a healthy and balanced diet, mainly as a rich source of vitamins, minerals and biologically active compounds. Despite all these benefits, the fruit microbiome also includes potential pathogens, such as viruses, bacteria and fungi, which may pose risk for humans health by decreasing the nutritive value and/or by producing injurious toxic metabolites [1]. In particular, soft fruits are sensitive to microbial contamination, involving both the fruits surface and tissues [2]. Filamentous fungi belonging to genera *Alternaria*, *Cladosporium*, *Penicillium*, *Fusarium* and *Aspergillus* are mainly responsible for the spoilage of fresh fruits [3, 4]. Infections acquired from exposure to food have been confirmed in case of *Mucor, Fusarium* and *Rhizopus*, occurred mostly among immunocompromised patients [5]. Fungi such as *Aspergillus* or *Penicillium* transmitted mainly by respiratory route, contribute to allergy, opportunistic infections or toxicosis [3, 6]. Cases of phytofungal infections, caused by *Alternaria*, *Cladosporium* and by some oomycete pathogens, have been also reported [7]. According to *Antimicrobial Resistance Global Report on Surveillance*, the harmful health effects of fungal infections are related with microbial resistance [8].

Antifungal resistance often appears as a result of using fungicides in agricultural ecosystems and developed as an inherited trait through natural selection process of fungi [9]. Fungal contamination of raw or minimally processed food may hold the risk for transfer of antifungal resistance to humans, similar to antibacterial resistance [10]. Current antifungal therapies are based on four groups of drugs: azoles, echinocandins, polyenes and pyrimidine analogs, however, azoles, echinocandins and polyenes are mainly applied in treatment of infections caused by filamentous fungi [11, 12].

The rapidly increasing level of drug resistance of microorganisms in the natural environment is a challenge for clinicians in terms of effective treatment of infections caused by filamentous fungi. Considering the significant role of fungal contaminants in food, both from the economic and public health point of view, the aim of the study was to determine the diversity, intensity and drug resistance of filamentous fungi isolated from the fresh raspberries.

A total of 41 samples of raspberries were collected randomly from croplands and home gardens during the summer and autumn periods in 2019–2020. The samples were analysed immediately after and collection and delivery to the laboratory. The comminuted fruits samples of 10 g each were suspended in 90 ml of Ringer's solution (Merck KGaA, Germany) and homogenized for 4 min with using the Bag Mixer 400 SW (Interscience, France). To determine the fungal population density, the plate dilution method on Malt Agar (Difco, USA) with the addition of chloramphenicol was used. Each inoculated dish was incubated at 30 °C for 72 h, then at room temperature (during the day and night at 22 °C in a laboratory room equipped with air conditioning) for further 72 h. Identification of filamentous fungi was based on macroscopic and microscopic methods with the use of keys and mycological atlases.

Susceptibility to antifungal drugs was determined by disc diffusion and gradient strip methods. The fungal inoculum suspensions comprising of mycelial bits and spores were prepared from 7-day old cultures grown on Malt Agar and the optical density was adjusted to a 0.5 McFarland standard. The antifungal agent discs (BioMaxima, Poland) used were: ketoconazole (10 μg), amphotericin B (20 μg), itraconazole (50 μg), caspofungin (5 μg), fluconazole (25 µg), posaconazole (5 µg), voriconazole (1 µg), flucytosine (1 µg) and nystatin (100 IU). The discs were placed on Malt Agar medium, and incubated at 30 °C for 72 h. The diameter of the inhibition zones was measured in millimeter (Fig. 1a). In gradient strips method, the minimum inhibitory concentration (MIC) was determined by the MIC Test Strip (Liofilchem, Italy): ketoconazole, amphotericin B, itraconazole, caspofungin, posaconazole, voriconazole, micafungin, anidulafungin, flucytosine (0,002–32 μg/ml) and fluconazole (0,016–256 μg/ml).The strips were placed on RPMI medium (BioMaxima, Poland) and incubated at 30 °C for 72 h. MIC was read directly from the scale in terms of µg/ml, at the point where the edge of the inhibition ellipse intersects with the strip (Fig. 1b).

**Fig. 1:**
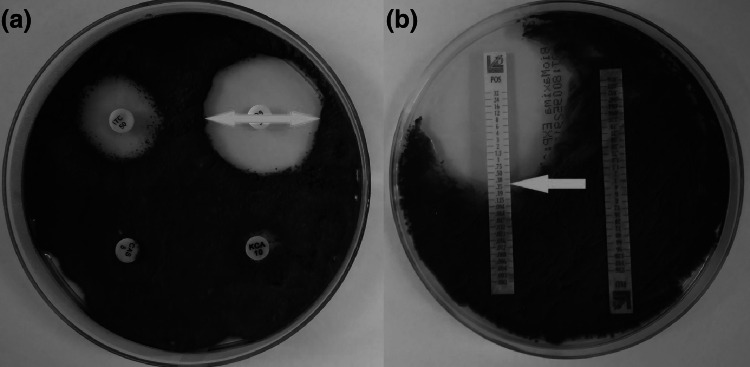
Measurement of the zone of inhibition the growth of fungi of the genus *Cladosporium* in the disc diffusion method (**a**) and the gradient stripes method (**b**)

The average mycological contamination in the examined samples amounted to the value of 4.34 log CFU/g (Table 1). The obtained median fungal concentration was similar to the results obtained by other researchers, with the values ranging from 4 log CFU/g to 6 log CFU/g [1]. *Cladosporium* could occur on raspberries at the early stage of produce storage, as well as during the storage, transport or sale stage [2]. Within this genus, the greatest number of the species was isolated from soil or plants. However, several species could affect animals and humans, including species mainly responsible for allergic rhinitis [13], such as *C. sphaerospermum* and *C. cladosporioides* isolated from raspberry fruits in this study. *Alternaria* and *Fusarium* genera were isolated from the fruits with prevalence at the level of 61% and 34.1%, respectively. Tournas and Katsoudas [14] confirmed the presence of *Alternaria* in 46% of tested blueberries and *Fusarium* in 25% of probes isolated from raspberries. The pathogenic influence of *Fusarium* on human health has been proved with reference to eye infections, mostly occurring in tropical and subtropical climate; however, infections have also been reported in the Netherlands [15]. The case of human ocular infection with *Alternaria infectoria* has also been confirmed after eye-perforating trauma caused by lemon tree branch [7].

**Table 1:** Qualitative and quantitative mycological composition of raspberries

Genus of filamentous fungi (species)	No. of contaminated samples (%)	Fungal concentration range [log CFU/g]	Mean + standard deviation [log CFU/g]	Median [log CFU/g]
*Cladosporium* (*C. cladosporioides, C. sphaerospermum*)	41 (100%)	1.74–4.88	3.58 (± 0.71)	3.68
*Alternaria* (*A. tenuissima, A. arborescens, A. infectoria*)	25 (61%)	0.02–1.16	0.35 (± 0.26)	0.25
*Fusarium* (*F. poae, F. culmorum, F. verticillioides, F. oxysporum*)	14 (34.1%)	0.04–0.56	0.27 (± 0.16)	0.26
*Rhizopus* (*R. oryzae*)	10 (24.4%)	0.04–0.35	0.14 (± 0.1)	0.1
*Penicillium* (*P. allii, P. expansum*)	6 (14.6%)	0.02–0.63	0.26 (± 0.23)	0.21
*Mucor* (*M. racemosus*)	6 (14.6%)	0.02–0.23	0.13 (± 0.07)	0.13
*Aspergillus* (*A. parasiticus, A. fumigatus, A. carbonarius)*	3 (7.3%)	0.02–0.28	0.15 (± 0.13)	0.16
*Aureobasidium*	4 (9.8%)	0.15–0.34	0.27 (± 0.09)	0.29
*Microsporum*	2 (4.9%)	0.03–0.04	0.04 (± 0)	0.04
*Trichoderma*	1 (2.4%)	0.49	0.49	0.49
*Acremonium*	1 (2.4%)	0.23	0.23	0.23
*Humicola*	1 (2.4%)	0.1	0.1	0.1
Non-sporulating fungi	34 (82.9%)	0.02–1.32	0.37 (± 0.28)	0.26
Total samples	41 (100%)	3.48–5.13	4.34 (± 0.39)	4.36

Among others potentially pathogenic fungi with the ability to produce mycotoxins, *Rhizopus* (24.4%), *Mucor* (14.6%), *Penicillium* (14.6%) and *Aspergillus* (7.3%) were isolated from the tested raspberries. The prevalence of these genus has been also confirmed on blueberries, strawberries and grapes [14]. The large group of mycological contamination were non-sporulating fungi identified in almost 83% of examined samples, mainly responsible for respiratory tract infections [16].

All fungal strains isolated from the raspberries showed 100% resistance to caspofungin, fluconazole and flucytosine in both methods and 100% resistance to micafungin, flucytosine and anidulafungin in the gradient strips method (Table 2 and 3). The highest drug resistance was observed in the case of *Acremonium* spp. (on 10 out of 11 tested antibiotics with the exception of the nystatin). In contrast to the strains isolated from environmental sources, the effectiveness of selected antibiotics, such as posaconazole and voriconazole against *Acremonium* species isolated from clinical samples, has been proved by in vitro studies [17]. Among the genus *Fusarium,* the greatest variation occurred in drug resistance among individual strains isolated from raspberries. As indicated by Sav et al*.* [11], the variation could be related with the ability of particular *Fusarium* strains to form biofilms, which may significantly increase the resistance. Antifungal susceptibility among clinical samples has been confirmed to amphotericin B, with MICs mostly ranging from 0.5–4 µg/ml [18], at the same level as in the present study. Al-Hatmi et al*.* [18] indicated that two of the most active drugs against *Fusarium* species were voriconazole and posaconazole, which is confirmed in this study (Table 3).

**Table 2:** Assessment of sensitivity to selected antifungal drugs among filamentous fungi isolated from raspberries using the disc diffusion method

Genera of fungi (number of strains)	Zone of inhibition [mm] Range (geometric mean; number of strains showing growth inhibition)								
	KCA	AMB	ITC	CAS	FLU	POS	VOR	AFY	NY
*Fusarium* (n = 10)	0–16 (11.7; n = 3)	0–11 (11; n = 1)	0–15 (12.2; n = 2)	rs*	rs	0–29 (23.3; n = 6)	0–20 (17.3; n = 5)	rs	0–25 (13.5; n = 8)
*Cladosporium* (n = 10)	0–21 (13.5; n = 6)	rs	15–30 (22.0; n = 10)	rs	rs	29–41 (32.9; n = 10)	rs	rs	17–23 (19.6; n = 10)
*Alternaria* (n = 10)	0–10 (10.0; n = 6)	rs	17–25 (18.5; n = 10)	rs	rs	28–32 (29.4; n = 10)	rs	rs	19–21 (20.0; n = 10)
*Penicillium* (n = 10)	10–23 (16.4; n = 7)	rs	23–35 (26.5; n = 7)	rs	rs	12–45 (30.4; n = 10)	10–18 (13.2; n = 6)	rs	13–28 (17.9; n = 10)
*Mucor* (n = 3)	rs	0–8 (8; n = 1)	0–13 (13; n = 1)	rs	rs	10–20 (15.0; n = 3)	rs	rs	20–21 (20.7; n = 3)
*Rhizopus* (n = 3)	12 (12; n = 3)	0–8 (8; n = 1)	18–20 (19.3; n = 3)	rs	rs	25 (25; n = 3)	rs	rs	23 (23; n = 3)
*Aspergillus* (n = 3)	0–20 (20; n = 3)	rs	22–25 (23.0; n = 3)	rs	rs	33–35 (34.3; n = 3)	10–15 (12.2; n = 3)	rs	17–20 (18.3; n = 3)
*Acremonium* (n = 1)	rs	rs	rs	rs	rs	rs	rs	rs	rs

^*^rs—fungi resistant to antifungal drugs (no zone of inhibition of growth)

Anti-fungal discs: ketoconazole (KCA), amphotericin B (AMB), itraconazole (ITC), caspofungin (CAS), fluconazole (FLU), posaconazole (POS), voriconazole (VOR), flucytosine (AFY) and nystatin (NY)

**Table 3:** Assessment of sensitivity to selected antifungal drugs among filamentous fungi isolated from raspberries using the gradient strip method

Genera of fungi (number of strains)	MIC value [μg/ml] Range (geometric mean; number of strains showing growth inhibition)									
	KCA	AMB	ITC	CAS	FLU	POS	VOR	MI	ANI	AFY
*Fusarium* (n = 10)	Rs*	0.5–4 (2; n = 3)	rs	rs	rs	3–8 (5.1; n = 5)	0.50–4 (1.3; n = 7)	rs	rs	rs
*Cladosporium* (n = 10)	0.25–1.50 (0.9; n = 10)	1–3 (1.8; n = 10)	0.25–3 (1.0; n = 10)	rs	rs	0.094–0.50 (0.3; n = 10)	0.25–3 (1.3; n = 10)	rs	rs	rs
*Alternaria* (n = 10)	2–4 (3.2; n = 3)	0.50–2 (1.2; n = 10)	4–12 (7.6; n = 9)	rs	rs	0.75–2 (1.3; n = 10)	1.50–12 (4.6; n = 6)	rs	rs	rs
*Penicillium* (n = 10)	0.19–8 (2.1; n = 7)	0.50–4 (1.4; n = 5)	1–24 (3.9; n = 7)	rs	rs	0.125–2 (0.6; n = 7)	0.094–3 (0.7; n = 6)	rs	rs	rs
*Mucor* (n = 3)	rs	1.5–4 (2.3; n = 3)	rs	rs	rs	6–8 (6.9; n = 2)	rs	rs	rs	rs
*Rhizopus* (n = 3)	6–8 (6.6; n = 3)	0.094–0.19 (0.15; n = 3)	16 (16; n = 3)	rs	rs	4–6 (5.8; n = 3)	rs	rs	rs	rs
*Aspergillus* (n = 3)	3–12 (6; n = 2)	0.75–3 (1.3; n = 3)	2–4 (2.9; n = 3)	rs	rs	0.38–0.50 (0.4; n = 2)	0.38–2 (0.7; n = 3)	rs	rs	rs
*Acremonium* (n = 1)	rs	rs	rs	rs	rs	rs	rs	rs	rs	rs

^*^rs—fungi resistant to antifungal drugs (no zone of inhibition of growth)

Gradient strips: ketoconazole (KCA), amphotericin B (AMB), itraconazole (ITC), caspofungin (CAS), fluconazole (FLU), posaconazole (POS), voriconazole (VOR), micafungin (MI), anidulafungin (ANI) and flucytosine (AFY)

Particularly noteworthy are the fungi belonging to the *Aspergillus*, which can cause life-threatening infections, especially in people with the weakened immune systems [9]. *Aspergillus* strains isolated from raspberries showed complete resistance to caspofungin, fluconazole, micafungin, anidulafungin and flucytosine. The rapid development of multi-drug resistance noticed in the case of *A. fumigatus* was the reason for placement of this species on the watch list within the report on antimicrobial resistance of the Centers for Disease Control and Prevention in the USA in 2019 [6].

Among different classes of drugs belonged to echinocandins, azoles, polyenes and pyrimidine analogues used in this study, the wide tolerance spectrum of tested fungal strains has been confirmed in at least two of them. According to many researches, the high antifungal resistance is related with the great number of pesticides used nowadays used in agriculture [9]. As a consequence, prolonged fungicide exposure may result in the occurrence of many more multi-drug-resistant fungal strains in fruits, vegetables and other crops, affecting both human health and food safety. Contamination of food with drug resistant fungi (especially multi-drug resistant) carries a risk of environmental resistance transmission to humans, reducing the treatment efficacy of potential human infections [8].

Taking into consideration that the most of filamentous fungi isolated from plants could be opportunistic pathogens to humans and the significant increase in the availability and widespread use of drugs, this work may be useful for further clinical research. The obtained results may also provide useful information to develop novel methods against fungal contamination for fruit at the early stage of production, during storage, transport and sale stage, as well as to determine appropriate standards and regulations for improving food safety, thereby limiting the risk of exposure to humans’ health.
